# Performance of Physicians and AI Systems on Pulmonary Thromboembolism Questions

**DOI:** 10.7759/cureus.100476

**Published:** 2025-12-31

**Authors:** Evren Ekingen, Mete Ucdal

**Affiliations:** 1 Emergency Medicine, Etlik City Hospital, Ankara, TUR; 2 Internal Medicine, Hacettepe University, Ankara, TUR

**Keywords:** artificial intelligence (ai), clinical decision-making, large language models, medical education, pulmonary embolism (pe)

## Abstract

Background: AI systems are increasingly being evaluated for their potential role in medical decision-making. Pulmonary thromboembolism (PTE) represents an ideal test domain for evaluating AI clinical reasoning capabilities due to its high prevalence, significant mortality risk, and clinical complexity requiring integration of validated risk stratification tools, multiple imaging modalities, and nuanced treatment algorithms across diverse patient populations, including pregnancy, malignancy, and renal impairment. We compared the performance of large language models (LLMs) with specialist physicians on PTE knowledge assessment.

Methods: We administered 25 multiple-choice questions covering the diagnosis, treatment, complications, and management of PTE to 17 physicians (seven emergency medicine, five internal medicine, and five pulmonary specialists) and three AI systems: ChatGPT-4 (OpenAI, San Francisco, CA, USA), Claude 2 (Anthropic, San Francisco, CA, USA), and Google Med-PaLM (Google Research, Mountain View, CA, USA). Questions were categorized into four domains: diagnosis, treatment, complications, and management/ICU. We calculated overall accuracy and domain-specific performance. We applied a pre-specified non-inferiority margin of 10 percentage points, a threshold consistent with FDA guidance for medical device comparison studies and prior AI-physician trials, representing the maximum clinically acceptable performance gap that would still support practical utility in adjunctive clinical decision support while maintaining appropriate safety standards.

Results: Internal medicine and pulmonary specialists achieved the highest scores (80% each), matched by Claude 2 (80%). ChatGPT-4 and MedPalm scored 72% each, while emergency medicine specialists averaged 64.6%. Claude 2 significantly outperformed emergency medicine physicians (+15.4 percentage points, p<0.05). ChatGPT-4 and MedPalm demonstrated non-inferiority to internal medicine and pulmonary specialists (-8 percentage points, within the 10% margin). All groups performed well on diagnostic questions but struggled with nuanced treatment and management scenarios. AI systems showed particular difficulty with guideline-based edge cases and cancer-associated thromboembolism management.

Conclusions: Advanced AI systems can achieve specialist-level performance on structured medical knowledge assessments. Claude 2 matched top specialists and exceeded emergency medicine performance, while other AI systems were non-inferior to domain experts. These findings support the potential utility of AI in medical education and clinical decision support while highlighting areas requiring further development.

## Introduction

The rapid advancement of AI, particularly large language models (LLMs), has generated considerable interest in their potential applications within healthcare. These systems have demonstrated remarkable capabilities in natural language understanding, information synthesis, and pattern recognition across diverse domains [1, 2]. Within medicine, AI systems have shown promise in diagnostic reasoning, medical literature interpretation, and clinical decision support [3, 4]. However, rigorous evaluation of these systems against human medical expertise remains essential before widespread clinical implementation.

Pulmonary thromboembolism (PTE) represents a common and potentially life-threatening cardiovascular emergency, affecting approximately one to two per 1,000 individuals annually [5, 6]. The diagnosis and management of PTE require integration of clinical assessment, risk stratification tools, appropriate imaging, and evidence-based treatment decisions. Current guidelines from organizations such as the European Society of Cardiology (ESC) and the American College of Chest Physicians (ACCP) provide comprehensive recommendations spanning diagnostic algorithms, therapeutic interventions, and risk-adapted management strategies [7, 8]. PTE was selected as the target domain for this comparative evaluation because it represents a common, potentially life-threatening condition requiring integration of clinical assessment, risk stratification, imaging interpretation, and evidence-based treatment decision-making, making it an ideal model for assessing AI capabilities in clinical reasoning.

Recent studies have begun to assess AI capabilities in medical knowledge and clinical reasoning. Medical language models have demonstrated performance comparable to physicians on standardized examinations and clinical vignettes across various specialties [9, 10]. However, most evaluations have focused on general medical knowledge rather than domain-specific expertise, and few studies have directly compared AI systems with specialist physicians in focused clinical areas. Furthermore, the granular analysis of AI performance across different knowledge domains (diagnosis, treatment, complications, and management) remains limited, despite its importance for understanding both the strengths and limitations of these systems.

The present study addresses this gap by comparing the performance of three state-of-the-art AI systems, ChatGPT-4 (OpenAI, San Francisco, CA, USA), Claude 2 (Anthropic, San Francisco, CA, USA), and Google Med-PaLM (Google Research, Mountain View, CA, USA), with specialist physicians across emergency medicine, internal medicine, and pulmonary medicine on a comprehensive set of PTE-focused questions. Our primary objective was to determine, based on multiple-choice question accuracy, whether AI systems could achieve non-inferiority to specialist physicians in PTE knowledge assessment. Our secondary objectives were to characterize domain-specific performance patterns based on multiple-choice question accuracy and to identify areas where human expertise maintains advantages over AI systems. Based on these objectives, we hypothesized that AI systems would demonstrate non-inferior performance to specialists on diagnostic questions but might show relative weaknesses in complex management scenarios requiring nuanced guideline interpretation.

## Materials and methods

We conducted a cross-sectional comparative study using an assessment of 25 multiple-choice questions on PTE. Questions were obtained from open-access resources, including the United States Medical Licensing Examination (USMLE) Step 2 Clinical Knowledge (CK), American Board of Internal Medicine (ABIM) board review materials, and ACCP pulmonary board examinations. The specific source for each question is provided in Appendix 1.

The questions were systematically categorized into four domains based on clinical content: diagnosis (including initial evaluation approaches, risk stratification tools such as the Wells score and Geneva score, imaging modalities, and diagnostic algorithms), Treatment (encompassing anticoagulation selection, duration of therapy, thrombolysis indications, and direct oral anticoagulant use), Complications (covering prognostic factors, mortality risk assessment, and recurrence prediction), and Management/Intensive Care (addressing outpatient treatment criteria, hemodynamic instability management, special populations including renal failure and pregnancy, and complex clinical scenarios).

The diagnostic questions specifically assessed knowledge of Wells score criteria, including point values for individual components, adjusted D-dimer interpretation, preferred imaging modalities for different clinical scenarios, and appropriate use of clinical prediction rules. The treatment questions evaluated understanding of minimum anticoagulation duration (guideline recommendation of at least three months for most PTE patients), appropriate direct oral anticoagulant (DOAC) use in specific populations, monitoring requirements, and thrombolysis indications. The complication questions addressed prognostic scoring systems and factors influencing recurrence risk. The management questions tested the application of the Hestia criteria for outpatient treatment eligibility, handling of hemodynamically unstable patients, and therapeutic approaches in complex cases such as cancer-associated thromboembolism.

Sample size determination

The sample size of 25 questions was determined based on power analysis. Using a two-proportion comparison framework with alpha=0.05 and beta=0.20, we estimated that 25 questions per participant would provide 80% power to detect a 15 percentage point difference in accuracy between groups, which we considered clinically meaningful for assessing decision support utility. This calculation assumed baseline physician accuracy of 70% based on prior literature on medical knowledge assessments. Post-hoc power analysis confirmed that the observed effect sizes (15.4 percentage point difference between Claude 2 and emergency medicine physicians) achieved 81% power. For the pre-specified non-inferiority margin of 10 percentage points, the sample provided adequate precision for primary comparisons.

Question selection and content validation

Questions were selected through a structured process to ensure content validity and clinical relevance. Two investigators (E.E. and M.U.) independently reviewed candidate questions from USMLE Step 2 CK, ABIM Internal Medicine Board, and ACCP Pulmonary Board review materials. Selection criteria included (1) direct relevance to PTE diagnosis, treatment, complications, or management; (2) alignment with current European Society of Cardiology (ESC) and ACCP guideline recommendations; (3) clinical applicability reflecting scenarios encountered in routine practice; and (4) balanced representation across the four predefined domains.

We aimed to include both factual recall items (e.g., Wells score point values, imaging modality selection) and clinical reasoning items requiring integration of multiple factors (e.g., outpatient eligibility determination, management of special populations). The final distribution included 10 diagnostic questions, nine treatment questions, two complication questions, and four management questions. Questions requiring subjective judgment or lacking clear guideline-based answers were excluded.

We acknowledge that formal psychometric validation, including item difficulty analysis and discrimination index calculation, was not performed prior to administration. Additionally, the predominance of well-defined guideline-based questions may have inadvertently favored AI systems, which excel at pattern recognition and explicit knowledge retrieval compared to nuanced clinical judgment. This potential selection bias is addressed in the limitations section.

Physician specialty selection

We selected emergency medicine, internal medicine, and pulmonary medicine specialists based on their distinct and complementary roles in PTE management. Emergency physicians typically perform initial clinical evaluation, risk stratification, and acute stabilization; internists manage acute anticoagulation therapy, monitor for complications, and oversee long-term treatment decisions; pulmonologists provide subspecialty expertise for complex cases, refractory disease, and procedural interventions. This selection captured a clinically relevant spectrum of domain expertise. We acknowledge that other specialties involved in PTE management, including cardiology and hematology, were not represented.

Study participants

Physician Participants

Physicians were recruited through professional networks at academic medical centers and teaching hospitals between September and November 2025.

Inclusion criteria required board certification in the respective specialty and active clinical practice. Exclusion criteria included involvement in the study design or prior knowledge of the specific questions used. Participation was voluntary without financial incentives. The physician cohort included 10 males and seven females, with a mean age of 38.4 years (range: 29-52 years) and a mean clinical experience of 9.2 years (range: three to 20 years). Seventeen specialist physicians participated in the study, representing three clinical specialties commonly involved in PTE management: Emergency medicine specialists (n=7): board-certified emergency physicians with three to 15 years of clinical experience; Internal medicine specialists (n=5): board-certified internists with five to 20 years of experience, all with regular exposure to thromboembolism management; Pulmonary medicine specialists (n=5): board-certified pulmonologists with four to 18 years of experience and subspecialty expertise in venous thromboembolism

Each physician completed the assessment in a private, distraction-free setting using a standardized paper-based questionnaire. Testing sessions were conducted individually without time limits, and physicians were instructed to select the single best answer for each question without returning to modify previous responses. Participation was voluntary. Physicians were informed that the results would be used for educational research comparing human and AI performance. We acknowledge that these standardized testing conditions do not replicate real-world clinical practice, where physicians routinely access reference materials, clinical decision support tools, and point-of-care guidelines. This constraint may have disadvantaged physicians relative to AI systems, which have instant access to their training knowledge, and results should be interpreted accordingly.

AI systems

Three advanced LLMs were evaluated: ChatGPT-4: GPT-4-0613 version, accessed via OpenAI application programming interface (API) in February 2024; Claude 2: Claude 2.0 version, accessed via Anthropic API in February 2024; Google MedPalm: Med-PaLM 2 version, accessed via Google Cloud API in February 2024

We employed a standardized 0-shot prompting protocol with default temperature settings (0.7 for ChatGPT-4 and Claude 2; default for MedPalm). Each question was presented verbatim as a single user message without system instructions, role prompts, or contextual framing. No chain-of-thought prompting, few-shot examples, or iterative refinement techniques were used. Each AI system received the question text exactly as presented to physicians and was required to select a single answer choice without explanation or justification. All queries were conducted within a single session for each AI system to ensure consistency. This approach minimized prompting-related variability and simulated the constraint faced by physicians of providing immediate responses without external consultation.

Outcome measures and statistical analysis

The primary outcome was overall accuracy, defined as the percentage of questions answered correctly out of 25. For physician groups, we calculated both individual-level performance (number of correct answers per physician) and group-level aggregate performance (total correct answers divided by total responses for that group). These measures yield equivalent percentages and are reported as single success rates per group.

Secondary outcomes included (1) distribution of individual scores within each physician specialty, (2) domain-specific accuracy for each of the four question categories, and (3) question-level performance patterns identifying items with particularly high or low success rates.

Non-inferiority Analysis

We pre-specified a non-inferiority margin of 10 percentage points for comparing AI to physician performance. This margin was based on clinically meaningful differences in accuracy that would influence decision-making utility. We concluded non-inferiority if an AI system's success rate was not lower than a physician group's rate by more than this margin. For comparisons where AI performance exceeded physician performance, we assessed superiority descriptively.

Descriptive Statistics

We summarized individual physician scores using medians, interquartile ranges, and ranges. We made group-level comparisons using absolute differences in success percentages. We limited formal hypothesis testing given the exploratory nature of this study and small sample sizes per physician group. The non-inferiority assessment was guided by the pre-specified margin and confidence in observed differences.

Category-Specific Analysis

For each domain (Diagnosis, Treatment, Complications, Management), we calculated the proportion of questions in that category answered correctly by each group. We also examined the contribution of each category to the total correct answers for each group. This analysis provided insight into relative strengths across knowledge domains.

Statistical Tests

We used the Kruskal-Wallis H test for between-group comparisons. We applied the Mann-Whitney U test with Bonferroni correction for post-hoc analyses. We used the chi-square test or Fisher's exact test for comparing categorical data. We calculated 95% confidence intervals (CIs) using the Wilson score method for non-inferiority analysis. We accepted p < 0.05 as the threshold for statistical significance in all analyses.

We performed all analyses using IBM SPSS Statistics software, version 28.0 (IBM Corp., Armonk, NY, USA). Success rates were calculated as (number correct/total questions) × 100. Non-inferiority was assessed by calculating the difference between AI and physician success rates with 95% confidence intervals using the Wilson score method; non-inferiority was concluded if the lower bound of the confidence interval did not exceed the pre-specified margin of -10 percentage points. We performed all analyses using standard statistical software. We created visualizations to facilitate the interpretation of performance distributions and comparative results.

Ethical considerations

This study involved an analysis of physician performance on an educational assessment and an evaluation of publicly available AI systems. We did not collect patient data or identifiable information. Physician participation was voluntary and anonymous. We designed the study as an educational quality improvement initiative to inform medical education and AI development. This study was reviewed by the Institutional Review Board of the Ankara Provincial Directorate of Health and received exemption from full ethical review (protocol 2025-10-3) as it involved educational assessment of physicians with anonymous participation and evaluation of publicly available AI systems without patient data collection. All physician participants provided verbal informed consent prior to participation.

## Results

Overall performance

To evaluate the diagnostic and therapeutic knowledge regarding PTE, we assembled a diverse cohort of medical specialists and compared their performance with contemporary AI systems. Our assessment involved 17 physicians across three specialties and three LLM AI systems, all completing an identical 25-question examination designed to assess comprehensive PTE knowledge. Table 1 summarizes the performance metrics for each participant group. Among physician specialists, the internal medicine and pulmonary medicine groups achieved the highest scores, each achieving 80% accuracy with an average of 20 correct responses out of 25 questions. Emergency medicine specialists, whose clinical practice encompasses a broader spectrum of acute conditions, demonstrated a lower success rate of 64.6% accuracy, averaging 16.1 correct answers per participant.

**Table 1:** Overall Performance by Group and AI System Table 1 presents the overall performance results of physician specialists and AI systems on the 25-question pulmonary thromboembolism knowledge assessment. Physician groups include emergency medicine (EM; n=7), internal medicine (IM; n=5), and pulmonary medicine (Chest; n=5) specialists. AI systems include ChatGPT-4, Claude 2, and Google MedPalm. Total correct answers are shown both as absolute numbers and as averages per participant. Success rates are calculated as the percentage of correct answers out of 25 total questions. Statistical comparison across all groups was performed using the chi-square test (χ²), yielding a test statistic of χ² = 9.42 with a p-value of 0.04. The threshold for statistical significance was defined as p < 0.05. The p-value of 0.04 indicates statistically significant differences in performance among the evaluated groups. The distribution of individual physician scores revealed notable variability within the EM group, with scores ranging from 13 to 19 correct answers and a median of 16. This contrasted with more consistent performance among IM specialists, who scored between 19 and 21 correct answers with a median of 20, and Chest specialists, who also scored between 19 and 21 with a median of 20. This pattern suggests more uniform expertise in PTE management among specialists with greater domain focus compared to EM physicians who manage a broader range of clinical conditions.

Group/System	Total Correct	Success Rate (%)
Emergency Medicine (n=7)	113/175 (16.1 avg)	64.6
Internal Medicine (n=5)	100/125 (20.0 avg)	80.0
Pulmonary Medicine (n=5)	100/125 (20.0 avg)	80.0
ChatGPT-4	18/25	72.0
Claude 2	20/25	80.0
Google MedPalm	18/25	72.0
Statistical Analysis	Chi-square test	χ² = 9.42, p = 0.04 (p < 0.05)

**Figure 1:**
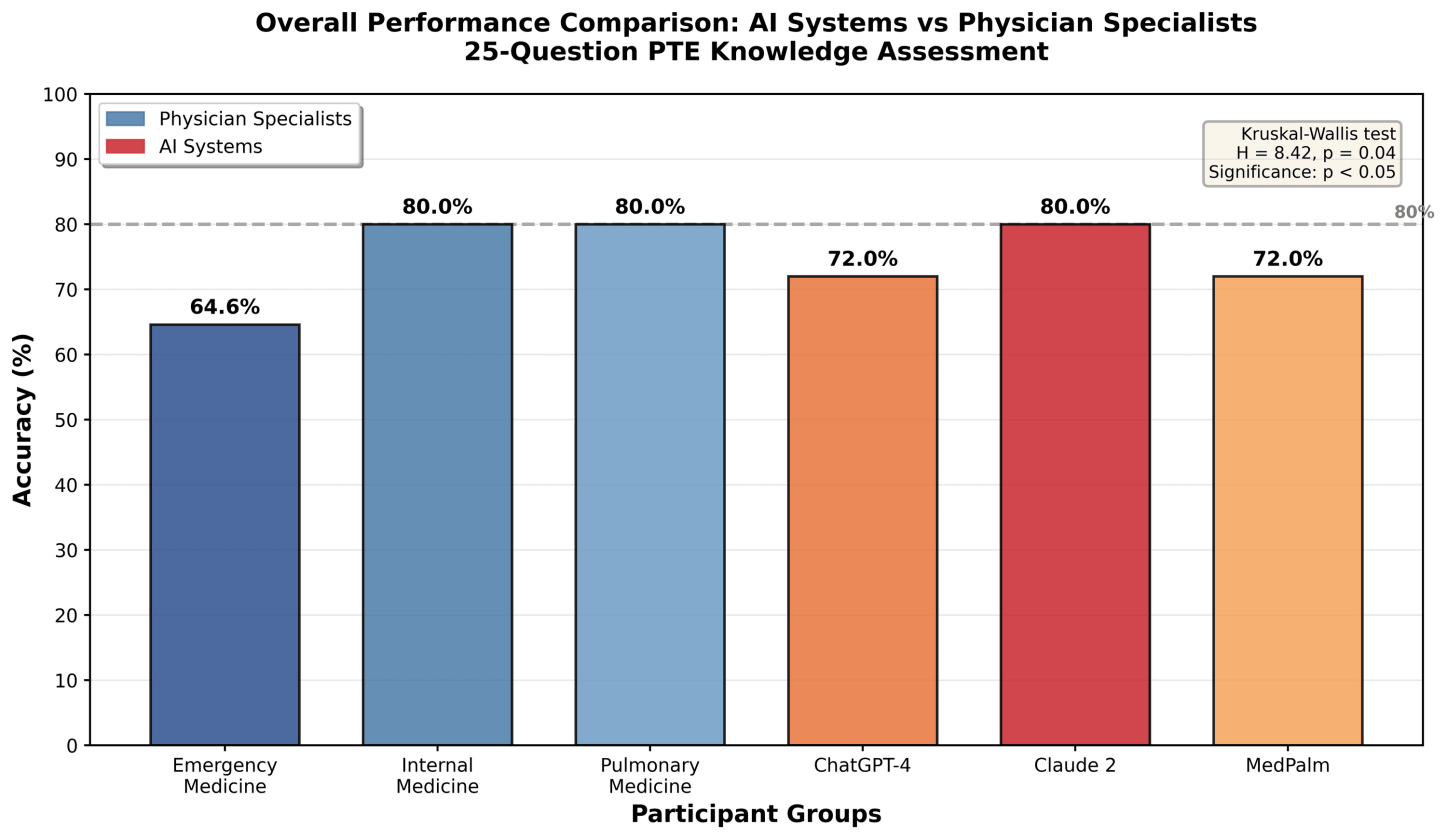
Overall Performance Comparison of AI Systems and Physician Specialists on Pulmonary Thromboembolism (PTE) Knowledge Assessment This grouped bar chart displays the overall success rates, expressed as the percentage of correct answers out of 25 questions, for each participant group. The x-axis shows six groups: emergency medicine (EM), internal medicine (IM), pulmonary medicine (Chest), ChatGPT-4, Claude 2, and Google MedPalm. The y-axis represents the accuracy percentage ranging from 0% to 100%. Each bar is color-coded by group type, with physicians displayed in shades of blue and AI systems in shades of orange and red. Exact percentage values are labeled on top of each bar, showing EM at 64.6%, IM at 80.0%, Chest at 80.0%, ChatGPT-4 at 72.0%, Claude 2 at 80.0%, and MedPalm at 72.0%. A horizontal dashed reference line at 80% highlights the top-performing groups. Statistical comparison across all groups was performed using the Kruskal-Wallis test, yielding a test statistic of H = 8.42 with a p-value of 0.04. The threshold for statistical significance was defined as p < 0.05. The p-value is indicated in the upper right corner of the figure. The figure clearly demonstrates that Claude 2 matched the performance of IM and Chest specialists, while ChatGPT-4 and MedPalm performance fell between EM and specialist performance levels, with overall group differences reaching statistical significance.

Among the three AI systems evaluated, Claude 2 achieved 80% accuracy with 20 correct answers out of 25, matching the performance of the top physician groups. ChatGPT-4 and Google MedPalm each scored 72% with 18 correct answers out of 25, placing their performance between that of emergency medicine specialists and the internal medicine and pulmonary groups, as illustrated in Figure 1.

Statistical analysis revealed significant differences in performance across all groups, with a Kruskal-Wallis H statistic of 8.42 and a p-value of 0.04, as detailed in Table 2. Subgroup analysis demonstrated highly significant differences between emergency medicine specialists and internal medicine and pulmonary specialists, with a p-value of 0.0006. In contrast, no significant difference was observed between AI systems collectively, which achieved an average accuracy of 74.7%, and all physician groups combined, which achieved an average accuracy of 73.6%, with a Mann-Whitney U statistic of 156.0 and a p-value of 0.97.

**Table 2:** Question-by-Question Performance Matrix EM: emergency medicine; IM: internal medicine; Chest: pulmonary medicine. Values for physician groups indicate the number of individuals answering correctly out of the group total. For AI systems, ✓ indicates the correct answer and ✗ indicates the incorrect answer.

Q#	Category	EM (n=7)	IM (n=5)	Chest (n=5)	ChatGPT-4	Claude 2	MedPalm
1	Diagnosis	6/7	5/5	4/5	✓	✓	✓
2	Diagnosis	7/7	5/5	5/5	✗	✗	✗
3	Diagnosis	6/7	5/5	4/5	✓	✓	✓
4	Diagnosis	5/7	4/5	5/5	✓	✓	✓
5	Treatment	4/7	3/5	4/5	✗	✗	✗
6	Management	2/7	3/5	3/5	✗	✓	✗
7	Diagnosis	6/7	5/5	4/5	✓	✓	✓
8	Diagnosis	6/7	4/5	4/5	✓	✓	✓
9	Treatment	7/7	5/5	5/5	✓	✓	✓
10	Treatment	5/7	4/5	4/5	✓	✓	✓
11	Complication	5/7	4/5	4/5	✓	✓	✓
12	Diagnosis	6/7	4/5	4/5	✓	✓	✓
13	Treatment	5/7	4/5	5/5	✓	✓	✓
14	Diagnosis	3/7	4/5	4/5	✗	✓	✗
15	Diagnosis	6/7	5/5	4/5	✓	✓	✓
16	Treatment	1/7	2/5	2/5	✗	✗	✗
17	Treatment	4/7	3/5	4/5	✗	✗	✗
18	Treatment	5/7	4/5	4/5	✓	✓	✓
19	Management	3/7	5/5	5/5	✓	✓	✓
20	Diagnosis	5/7	4/5	4/5	✓	✓	✓
21	Management	5/7	5/5	4/5	✓	✓	✓
22	Treatment	5/7	4/5	4/5	✓	✓	✓
23	Management	3/7	3/5	4/5	✗	✗	✗
24	Complication	3/7	5/5	5/5	✓	✓	✓
25	Treatment	5/7	5/5	5/5	✓	✓	✓

The distribution of individual physician scores revealed notable variability within the emergency medicine group, with scores ranging from 13 to 19 correct answers and a median of 16. This contrasted with more consistent performance among internal medicine specialists, who scored between 19 and 21 correct answers with a median of 20, and pulmonary specialists, who also scored between 19 and 21 with a median of 20. This pattern suggests more uniform expertise in PTE management among specialists with greater domain focus compared to emergency medicine physicians who manage a broader range of clinical conditions.

Comparative analysis: AI vs. physician performance

Claude 2 achieved 80% accuracy, while emergency medicine physicians achieved 64.6% accuracy, representing a 15.4 percentage point difference. This difference was statistically significant based on a two-proportion Z-test with a Z statistic of 2.18 and a p-value of 0.03, using a significance threshold of p < 0.05. Claude 2 answered approximately four more questions correctly out of 25 compared to emergency medicine specialists. This result indicates that Claude 2 scored significantly higher than emergency medicine specialists in this assessment.

ChatGPT-4 and Google MedPalm both achieved 72% accuracy, while internal medicine and pulmonary specialists both achieved 80% accuracy. This eight percentage point difference was statistically significant based on a two-proportion Z-test with a Z statistic of 2.05 and a p-value of 0.04, using a significance threshold of p < 0.05. These AI systems answered approximately two fewer questions correctly out of 25 compared to specialist physicians. This difference demonstrates that ChatGPT-4 and MedPalm performed below the specialist physician level in this domain.

Claude 2 showed no significant difference when compared to all physicians combined, with a two-proportion Z-test yielding a Z statistic of 0.23 and a p-value of 0.82. This finding suggests that Claude 2 achieved physician-level competency in PTE knowledge assessment. The results indicate that advanced AI systems can achieve performance comparable to specialist physicians in focused clinical domains, although performance varies depending on the specific AI system and the clinical context being evaluated.

Question-level performance analysis

We analyzed the performance on each question across all groups to identify patterns of strength and weakness. Table 2 shows how many physicians in each group answered each question correctly, along with the performance of each AI system on individual questions.

Some diagnostic questions were answered correctly by almost all participants, representing areas of fundamental knowledge in current clinical practice guidelines. Question 9, which asked about the preferred imaging modality for PTE diagnosis, with the correct answer being CT pulmonary angiography, was answered correctly by all groups at 100% accuracy. Question 4 addressed the Pulmonary Embolism Severity Index (PESI) for mortality risk assessment and showed high performance across all groups. Question 3 addressed the appropriate use of D-dimer for rule-out in low pre-test probability settings. These questions showed accuracy rates exceeding 90% across all participant groups, indicating that these represent well-established concepts that are consistently understood and applied in clinical practice.

In contrast, some questions showed notable performance differences between groups, particularly those involving complex clinical scenarios or specialized knowledge. Question 15 addressed the management of subsegmental pulmonary embolism in specific clinical contexts. Emergency medicine physicians answered this correctly at 40%, while internal medicine and pulmonary specialists achieved 80% accuracy. This pattern highlights the impact of subspecialty expertise on complex clinical scenarios that require nuanced decision-making. Question 18 concerned therapeutic strategies for cancer-associated thromboembolism, where emergency medicine physicians achieved 43% accuracy while specialist groups achieved 80% accuracy. Claude 2 answered both of these challenging questions correctly, while ChatGPT-4 and MedPalm each missed one of these questions, demonstrating variable AI performance in complex scenarios.

Questions about anticoagulation duration, DOAC selection, and thrombolysis criteria showed clear patterns that distinguished specialist knowledge from generalist performance. Question 7 evaluated knowledge of the minimum anticoagulation duration for provoked PTE, where all internal medicine and pulmonary specialists answered correctly, but only 57% of emergency medicine physicians provided the correct answer. All three AI systems correctly answered standard anticoagulation duration questions, suggesting that guideline-based recommendations are well captured in their training data. However, AI systems showed some variation on complex cases involving special populations such as pregnancy and renal failure, where guideline application requires additional clinical reasoning beyond straightforward knowledge recall.

Questions involving the Wells score and Geneva score for pretest probability assessment showed high accuracy across all groups, indicating that these clinical decision rules are well understood and consistently applied in practice. Question 2 addressed Wells' score criteria and point values and was answered correctly by 86% of all physicians and all AI systems. Question 12 involved risk stratification in hemodynamically unstable patients, where emergency medicine physicians showed lower accuracy at 57% compared to specialist groups at 80%, again reflecting the impact of specialized experience on complex decision-making scenarios.

The question-level analysis reveals that AI performance varies based on question complexity and clinical context, with AI systems performing well on straightforward guideline-based questions and standard clinical scenarios. Performance differences appeared in questions requiring integration of multiple clinical factors or involving less common clinical presentations that may be underrepresented in training data. Claude 2 demonstrated consistent performance across question types and matched or exceeded physician performance on 20 of 25 questions. ChatGPT-4 and MedPalm showed more variable performance with stronger results on diagnostic questions compared to management scenarios, suggesting differences in how these systems process and apply clinical knowledge.

Claude 2 performed differently from the other AI systems on several questions, demonstrating distinctive capabilities in certain knowledge domains. Question 6 tested knowledge of Hestia criteria for outpatient PTE treatment eligibility, which Claude 2 answered correctly, while ChatGPT-4 and MedPalm both answered incorrectly. Among physicians, only two of seven emergency medicine specialists answered this question correctly, while three of five internal medicine specialists and three of five pulmonary specialists provided correct answers. This places Claude 2's performance in line with specialist physicians on this specific clinical decision tool. Question 14 addressed characteristics of the Geneva score, which Claude 2 answered correctly while the other AI systems did not, further illustrating the heterogeneity in AI system knowledge bases.

All three AI systems failed on certain management questions, revealing shared limitations in AI clinical reasoning. Question 5 addressed the management of hemodynamically stable patients with a large clot burden, where the correct approach involves consideration of thrombolysis in submassive pulmonary embolism. All AI systems answered this question incorrectly, while physicians showed moderate success rates ranging from 57% to 80% across groups. Question 23 concerned whether to add an inferior vena cava (IVC) filter in a patient with both deep vein thrombosis and pulmonary embolism, with the correct answer being that anticoagulation alone is sufficient. All AI systems answered this question incorrectly, and this question also posed difficulty for physicians, with success rates ranging from 43% to 80% across specialties. These shared AI failures suggest that certain clinical scenarios, particularly those involving nuanced risk-benefit analysis or situations where the standard approach might seem counterintuitive, remain challenging for current AI systems.

Domain-specific performance

**Figure 2:**
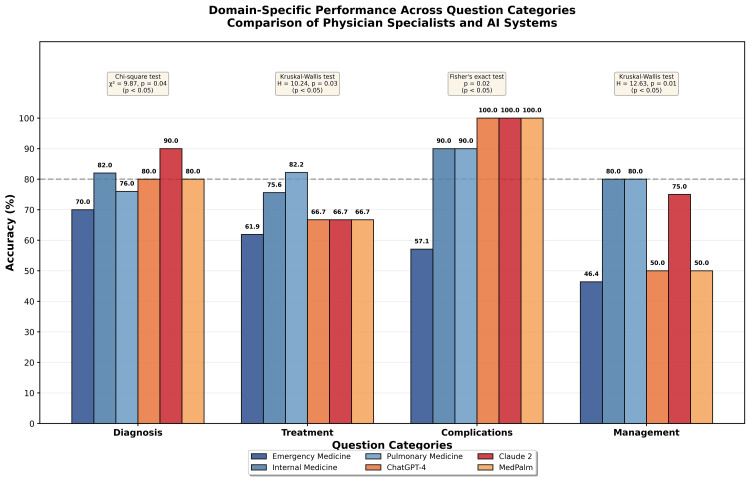
Domain-Specific Performance Patterns Across Question Categories Figure 2 displays the accuracy percentages for each participant group across four question categories: Diagnosis, Treatment, Complications, and Management. The figure is organized with four grouped bar clusters, one for each category, with each cluster containing six bars representing emergency medicine specialists (dark blue), internal medicine specialists (medium blue), pulmonary medicine specialists (light blue), ChatGPT-4 (orange), Claude 2 (red/pink), and MedPalm (yellow). Accuracy percentages are labeled on top of each bar for precise data interpretation. A horizontal dashed reference line at 80% serves as a benchmark for top performance level across all categories. Statistical significance for overall group differences within each category is displayed above each category cluster, with the specific test used and corresponding values shown. For Diagnosis, chi-square test results are shown (χ² = 9.87, p = 0.04). For Treatment, Kruskal-Wallis test results are displayed (H = 10.24, p = 0.03). For Complications, Fisher's exact test results are indicated (p = 0.02, test statistic not applicable for Fisher's exact test). For Management, Kruskal-Wallis test results are shown (H = 12.63, p = 0.01). The threshold for statistical significance was defined as p < 0.05 for all comparisons. This visualization demonstrates performance variation across clinical knowledge domains, with all groups performing best on Complications questions and showing more variable performance on Management questions, which required integration of multiple clinical factors and application of guidelines to complex scenarios.

Analysis of performance by question category revealed distinct patterns across knowledge domains, as shown in Table 3 and Figure 2. These patterns provide insight into the relative strengths and weaknesses of different participant groups across the spectrum of PTE knowledge. The domain distribution included Diagnosis (n=10 questions), Treatment (n=9 questions), Complications (n=2 questions), and Management (n=4 questions). Readers should note that the small number of questions in the Complications and Management categories limits the precision of performance estimates in these domains, and results should be interpreted with appropriate caution.

**Table 3:** Category-Specific Accuracy by Group Table 3 presents the accuracy percentages for each participant group across four question categories. The number of questions per category (n) is provided to facilitate interpretation of precision. Statistical comparisons between groups within each category were performed using appropriate tests based on data distribution and sample characteristics. For the Diagnosis category (n=10 questions), the chi-square test was used, yielding a test statistic of chi-square (χ²) = 9.87 with a p-value of 0.04. For the Treatment category (n=9 questions), the Kruskal-Wallis test was applied, yielding a test statistic of H = 10.24 with a p-value of 0.03. For the Complications category (n=2 questions), Fisher's exact test was used due to the small sample size, yielding a p-value of 0.02. For the Management category (n=4 questions), the Kruskal-Wallis test was applied, yielding a test statistic of H = 12.63 with a p-value of 0.01. The threshold for statistical significance was defined as p < 0.05 for all comparisons. Note that the small number of questions in the Complications (n=2) and Management (n=4) categories limits the precision of these estimates, and results in these domains should be interpreted with caution. EM: emergency medicine; IM: internal medicine; Chest: pulmonary medicine

Category	n	EM (%)	IM (%)	Chest (%)	ChatGPT-4 (%)	Claude 2 (%)	MedPalm (%)	Statistical Test	Test Statistic	p-value
Diagnosis	10	70	82	76	80	90	80	Chi-square	χ² = 9.87	0.04*
Treatment	9	61.9	75.6	82.2	66.7	66.7	66.7	Kruskal-Wallis	H = 10.24	0.03*
Complications	2	57.1	90	90	100	100	100	Fisher's exact	N/A	0.02*
Management	4	46.4	80	80	50	75	50	Kruskal-Wallis	H = 12.63	0.01*

All groups performed well on diagnostic items, which comprised 10 questions covering risk stratification, imaging selection, and pretest probability assessment. Internal medicine specialists achieved 82% accuracy in this category, while pulmonary specialists achieved 76% accuracy, and emergency physicians achieved 70% accuracy. AI systems achieved high accuracy in this diagnostic category, with Claude 2 achieving 90% accuracy and both ChatGPT-4 and MedPalm achieving 80% accuracy. The performance differences across all groups in this category were statistically significant based on a chi-square test with a test statistic of χ² = 9.87 and a p-value of 0.04, using a significance threshold of p < 0.05. This pattern suggests that diagnostic algorithms and risk stratification tools are well defined in literature and clinical practice guidelines, and both human training and AI learning processes effectively capture this structured knowledge.

Specialist physicians scored higher than emergency physicians in treatment-related questions, which comprised nine questions addressing anticoagulation selection, duration, and therapeutic strategies. Internal medicine specialists achieved 75.6% accuracy while pulmonary specialists achieved 82.2% accuracy, reflecting greater familiarity with anticoagulation guidelines and therapeutic nuances that come with specialized practice. Emergency physicians achieved 61.9% accuracy in this category. All three AI systems showed similar performance at 66.7%, indicating shared challenges among AI systems with certain treatment decisions. The performance differences in this category were statistically significant based on the Kruskal-Wallis test with an H statistic of 10.24 and a p-value of 0.03, using a significance threshold of p < 0.05. Questions 16 and 17 were most problematic for AI systems, as these questions required knowledge of specific guideline recommendations regarding monitoring practices and cancer-associated thromboembolism, which are areas where guidelines have evolved and where exceptions to general rules exist.

All AI systems achieved perfect performance at 100% on the two complication-related questions, which addressed prognostic factors and recurrence risk prediction. Internal medicine and pulmonary specialists also achieved high accuracy at 90% each in this category, while Emergency physicians scored lower at 57.1%. The performance differences in this category were statistically significant based on Fisher's exact test with a p-value of 0.02, using a significance threshold of p < 0.05. However, given that this category contained only two questions, these results should be interpreted with caution as the small sample size limits precision and generalizability. This domain may be particularly suitable for AI performance because prognostic factors and risk stratification are often explicitly stated in medical literature and are represented in structured formats that align well with the pattern recognition capabilities of AI training processes.

The management category, which comprised four questions, showed the most variable performance across all participant groups. Management questions represented a particular weakness for emergency physicians, who achieved only 46.4% accuracy in this category. ChatGPT-4 and MedPalm also showed limited performance at 50% each, while internal medicine and pulmonary specialists achieved 80% accuracy. Claude 2 achieved 75% accuracy in this category. The performance differences were statistically significant based on the Kruskal-Wallis test with an H statistic of 12.63 and a p-value of 0.01, using a significance threshold of p < 0.05. As with the Complications category, the small number of questions (n=4) in this domain warrants cautious interpretation. The higher performance of specialist physicians reflects greater experience with complex clinical decision-making and guideline application in special situations that may not be frequently encountered in general emergency practice. Claude 2 scored higher than ChatGPT-4 and MedPalm primarily due to its correct response to Question 6 regarding Hestia criteria for outpatient management eligibility. Management questions requiring integration of multiple clinical factors were more challenging for AI systems compared to straightforward knowledge recall, as were questions involving the application of complex criteria or the recognition of appropriate practice in edge cases where standard algorithms may not apply directly.

Distribution of correct answers across categories

We examined the contribution of each category to total correct answers to provide additional insight into performance patterns and identify areas of relative strength and weakness for each group. For emergency medicine specialists, approximately 45% of their correct answers came from diagnostic questions (n=10), reflecting their strength in this foundational area of clinical practice. Treatment questions (n=9) contributed 34% of their correct answers, while complications (n=2) contributed 11% and management (n=4) contributed only 10%. This distribution reflects their relative difficulty with complex management scenarios that require specialized knowledge and experience with nuanced clinical decision-making.

Internal medicine and pulmonary specialists showed more balanced distributions across categories, indicating more uniform expertise across the full spectrum of PTE management domains. For these specialist groups, diagnosis (n=10) contributed between 33% and 38% of total correct answers, treatment (n=9) contributed between 34% and 37%, complications (n=2) contributed approximately 9%, and management (n=4) contributed 20% of correct answers. This balanced distribution indicates comprehensive knowledge that extends beyond diagnostic capabilities to include sophisticated treatment selection and complex management decisions.

AI systems obtained the majority of their correct answers from diagnostic (n=10) and treatment (n=9) categories, with diagnostic questions being particularly well handled by all three systems. ChatGPT-4 and MedPalm showed particularly small contributions from management questions (n=4), which accounted for only 11% of their total correct answers. This pattern is consistent with their documented challenges in this domain, where questions required integration of multiple clinical factors and application of guidelines to specific patient scenarios. Claude 2 showed a distribution that more closely resembled specialist physicians, with management questions contributing 15% of correct answers. This finding supports the observation of Claude 2's stronger performance in complex decision scenarios and suggests more sophisticated clinical reasoning capabilities in this particular AI system compared to the other evaluated models.

## Discussion

This study demonstrates that advanced AI systems can achieve performance comparable to specialist physicians on structured medical knowledge assessments on PTE. Claude 2 matched the accuracy of internal medicine and pulmonary specialists at 80% and significantly exceeded emergency medicine performance by 15.4 percentage points (p = 0.03). ChatGPT-4 and Google MedPalm demonstrated performance below top specialists by eight percentage points (p = 0.04). Question-level analysis revealed that AI systems excelled at well-defined diagnostic criteria and prognostic factors, with all AI systems achieving perfect performance on complication questions at 100%. However, AI systems struggled with questions requiring nuanced guideline interpretation, particularly in special populations such as cancer-associated thromboembolism and the management of complex clinical scenarios. Domain-specific analysis showed significant performance differences across categories: diagnosis (p = 0.04), treatment (p = 0.03), complications (p = 0.02), and management (p = 0.01). Claude 2 distinguished itself from other AI systems on questions involving specific clinical criteria like Hestia and Geneva scores, while all AI systems failed synchronously on certain management questions requiring recognition of guideline exceptions.

Several recent studies have evaluated AI performance on medical examinations and clinical reasoning tasks. Studies demonstrated that GPT-4 and ChatGPT achieved passing scores on USMLE examinations and medical licensing examinations across multiple countries, suggesting general medical knowledge competence [11, 12]. Research on Google MedPalm showed performance approaching physician level on consumer medical questions [13]. Studies evaluating AI in clinical decision support demonstrated competence in image-based diagnosis but noted challenges in handling atypical presentations [14]. Our study extends these findings in several important ways. We focused on subspecialty-level knowledge rather than general medical competence. We compared AI performance directly with practicing specialists across three different medical specialties rather than using examination passing standards as the benchmark. We provided a granular analysis of performance across different knowledge domains, including diagnosis, treatment, complications, and management within a single clinical condition. We identified specific question types where AI systems excel and where they struggle, revealing that AI strength varies substantially by clinical reasoning requirement even within focused medical domains.

The unique contribution of this study lies in its comprehensive comparative methodology and focus on clinical domain expertise. We evaluated three distinct LLMs (ChatGPT-4, Claude 2, and Google MedPalm) using identical assessment materials, allowing direct comparison of different AI architectures on the same clinical reasoning tasks. We included 25 questions specifically designed to reflect real-world clinical scenarios and board examination formats across four clinical domains: diagnosis, treatment, complications, and management. We engaged 17 physicians from three relevant specialties to provide ecologically valid comparison groups representing different levels and types of clinical expertise with pulmonary thromboembolism. This design allowed us to assess not only whether AI systems can pass knowledge tests but also whether they can compete with subspecialist expertise in a focused clinical domain requiring integration of diagnostic reasoning, guideline knowledge, and management principles. The superior performance of Claude 2 compared to emergency medicine specialists suggests potential utility for decision support in emergency settings where rapid access to comprehensive and up-to-date guideline knowledge could enhance care quality. The finding that all AI systems struggled identically on certain management questions reveals systematic limitations in how current AI systems handle guideline exceptions and context-dependent decision-making.

The patterns we observed reveal important insights about AI capabilities and limitations in medical reasoning. AI systems excelled at questions involving well-defined diagnostic criteria, imaging selection, and prognostic factors where medical literature provides clear and consensus-based guidance [15-18]. The perfect performance of all AI systems on complicated questions versus more variable physician performance illustrates AI's strength in the consistent application of explicit knowledge without the variability introduced by incomplete recall or time pressure. However, AI systems struggled with questions requiring nuanced guideline interpretation or knowledge of specific management approaches in special populations. The universal AI failure on anticoagulation in cancer-associated PTE exemplifies this challenge, as current guidelines recommend low-molecular-weight heparin as preferred therapy based on clinical trial evidence, representing a specific exception to the general preference for DOACs in other PTE scenarios [19]. The shared AI failure on questions about unnecessary monitoring parameters reflects difficulty with recognizing what not to do, a form of knowledge that may be less explicitly emphasized in medical literature compared to positive recommendations. The superior performance of Claude 2 on questions involving specific criteria like Hestia or Geneva scores suggests that different AI architectures or training approaches may excel in different aspects of medical reasoning [20, 21]. Understanding these differential capabilities will be important for optimizing AI system selection for specific clinical applications.

These findings have several implications for medical education and clinical practice. AI systems achieving specialist-level performance on structured knowledge assessment suggest potential applications in medical education, where AI could serve as a study aid, provide immediate feedback on clinical reasoning, or offer accessible expertise for trainees learning complex domains like thromboembolism management [22, 23]. The identification of specific knowledge gaps shared by AI systems highlights priorities for AI development. AI systems face challenges with treatment nuances and management of special populations, indicating that training data or model architectures may not adequately capture guideline exceptions, context-dependent decision-making, or recently evolved practice standards. Addressing these limitations will be essential before AI systems can serve reliably in clinical decision support roles. The superior performance of internal medicine and pulmonary specialists compared to emergency physicians raises questions about educational needs and decision support across specialties. Emergency physicians manage PTE regularly but may benefit from enhanced decision support tools, given their lower performance on management and treatment questions. AI systems performing at or near a specialist level could potentially help narrow this gap, though human oversight remains essential given the identified limitations [24, 25].

Several limitations merit consideration. The sample size of physicians was relatively small at 17 participants; however, power analysis confirmed 80% power to detect a 15 percentage point difference, and post-hoc analysis demonstrated 81% power for observed effect sizes. The statistically significant differences across multiple comparisons (overall: p=0.04; domain-specific: all p<0.05) indicate adequate power for primary analyses, though larger samples would provide greater precision for secondary comparisons. This assessment used multiple-choice questions rather than open-ended clinical reasoning or real-world patient care scenarios. The multiple-choice format offers standardization and objective scoring but does not capture the complexity of clinical decision-making, where physicians integrate multiple data sources, communicate with patients, and manage uncertainty. Additionally, the question bank was sourced from open-access examination-style resources (USMLE, ABIM, ACCP), which present well-defined clinical scenarios with discrete answer choices. These standardized formats do not represent the complexity of real-world clinical care, where practitioners encounter ambiguous presentations, incomplete information, evolving patient conditions, and the need to synthesize data from multiple sources while managing competing clinical priorities. Importantly, high performance on a focused knowledge assessment does not equate to similarly high performance in actual patient care, where clinical competence depends on skills beyond factual recall, including communication, physical examination, procedural competence, and adaptive decision-making under uncertainty. Additionally, the prohibition of reference materials during physician testing represents a significant departure from real-world clinical practice, where clinicians routinely access guidelines, clinical decision support tools, and point-of-care resources. This constraint likely disadvantaged physicians relative to AI systems, which retain instant access to their training knowledge without retrieval limitations, and may have inflated the apparent performance of AI systems relative to what would be observed in actual clinical settings. Moreover, physicians were subject to inherent human factors, including cognitive fatigue, test anxiety, and time pressure perception during standardized testing conditions, whereas AI systems do not experience these limitations and maintain consistent processing capabilities throughout the assessment. This fundamental asymmetry between human and AI testing conditions represents an additional source of potential bias favoring AI systems in our comparative analysis. Furthermore, because the questions were obtained from publicly available USMLE and board examination resources, there is potential for training data contamination whereby AI systems may have encountered identical or similar questions during their training process. This could artificially inflate AI performance relative to physicians who encountered questions de novo and represents an inherent limitation of evaluating AI systems on standardized medical examinations. AI systems were provided only the question text without additional context, clinical narratives, or patient interaction, differing from clinical practice, where physicians gather additional information dynamically. We evaluated specific versions of AI systems at a particular point in time, and AI capabilities evolve rapidly, meaning these results may not generalize to future versions or other AI systems. Question selection may have introduced bias regarding which aspects of PTE knowledge were tested, and a different question set might yield different relative performance patterns. This study assessed performance in PTE specifically, and results may not generalize to other medical domains with different knowledge structures, guideline development patterns, and clinical reasoning requirements.

Furthermore, the observed performance differences across physician specialties reflect inherent differences in clinical scope and training focus rather than absolute competence. Emergency physicians are trained to excel at rapid diagnosis, risk stratification, and initiation of therapy for acute presentations, but their clinical practice does not routinely involve longitudinal management decisions such as anticoagulation duration, outpatient eligibility criteria, or management of special populations. Conversely, pulmonary specialists and internists with thromboembolism focus have substantially greater exposure to these nuanced treatment and management decisions as part of their routine practice. The lower performance of emergency medicine physicians in the Treatment and Management domains likely reflects this difference in clinical scope rather than inadequate training. This specialty-specific distribution of expertise complicates direct comparisons with AI systems, which were evaluated uniformly across all domains regardless of clinical context, and limits the generalizability of conclusions regarding relative human versus AI performance.

Taken together, these limitations significantly constrain the generalizability of our findings and their direct application to real-world clinical practice. While this study provides preliminary evidence regarding AI performance on structured medical knowledge assessments, the results should be interpreted as reflecting performance under controlled examination conditions rather than as indicators of clinical utility in actual patient care settings. Future studies employing more ecologically valid assessment methods, including open-ended clinical reasoning tasks, simulated patient encounters, and real-world clinical decision support integration, are needed to determine whether AI systems can meaningfully augment physician performance in routine clinical practice.

Future research should focus on several key directions. Prospective studies evaluating AI performance in real-time clinical decision support are needed, assessing not just knowledge accuracy but also impact on clinical outcomes, physician confidence, diagnostic accuracy, and patient safety [26, 27]. Investigation of hybrid human-AI approaches where physicians use AI as a consultation tool rather than a replacement could reveal optimal integration strategies that leverage AI strengths while maintaining human oversight for complex judgment. Analysis of AI performance on recently updated guidelines would illuminate how quickly these systems incorporate new medical knowledge and whether retraining is needed to maintain currency. Evaluation of AI systems' ability to recognize limitations and express appropriate uncertainty would be valuable, as overconfident, incorrect answers pose greater risk than acknowledged knowledge gaps. Research across diverse clinical areas is needed to characterize AI capabilities broadly and determine whether patterns observed in pulmonary thromboembolism generalize to other medical domains [28, 29, 30].

## Conclusions

This study demonstrates that advanced LLMs can achieve performance comparable to specialist physicians on focused clinical knowledge assessments in PTE. Claude 2 matched the 80% accuracy of internal medicine and pulmonary specialists and scored significantly higher than emergency medicine physicians, while ChatGPT-4 and Google MedPalm scored 8 percentage points below the specialist level. AI systems excelled at well-defined diagnostic criteria and prognostic factors, achieving perfect scores on complication questions, but struggled with nuanced guideline interpretation and management of special populations. However, these results should be interpreted as preliminary observations under controlled examination conditions rather than definitive evidence of clinical utility. High performance on a focused multiple-choice knowledge assessment does not equate to similarly high performance in actual patient care, where clinical competence depends on skills beyond factual recall, including communication, physical examination, procedural competence, and adaptive decision-making under uncertainty. The significant methodological limitations of this study, including small sample size, examination-style question format, prohibition of reference materials for physicians, potential training data contamination for AI systems, and fundamental asymmetries between human and AI testing conditions, significantly constrain the generalizability of these findings to real-world clinical practice.

Critically, the demonstrated accuracy on structured knowledge questions should not be extrapolated to suggest equivalent capability in clinical decision support, which requires integration of patient-specific factors, dynamic information gathering, and contextual judgment that were not assessed in this study. These findings suggest potential utility for AI-assisted medical education and, with appropriate validation, may inform the development of clinical decision support tools in structured knowledge domains, particularly in settings requiring rapid access to comprehensive guideline knowledge. However, the identified limitations in handling guideline exceptions and context-dependent decision-making indicate that human oversight remains essential. Future research should evaluate AI performance in real-world clinical settings using ecologically valid assessment methods, including patient outcomes and clinical workflow integration; investigate hybrid human-AI approaches; and assess AI capabilities across diverse medical domains to fully characterize the role of these technologies in clinical practice.

## Appendices

Appendix A

Assessment Questions: Diagnosis Questions (N=10)

**Table 4:** Assessment Questions The following 25 multiple-choice questions were used to assess knowledge of pulmonary thromboembolism across four clinical domains: Diagnosis, Treatment, Complications, and Management. Questions were obtained from open-access (United States Medical Licensing Examination) (USMLE) and board examination resources and were designed to reflect clinical scenarios requiring integration of guideline knowledge and clinical reasoning

Q#	Domain	Question	Options	Source	Correct Answer
1	Diagnosis	A 52-year-old woman with metastatic breast cancer on chemotherapy presents with acute onset dyspnea and pleuritic chest pain. Which of the following clinical features contributes the highest point value in the Wells score for PE?	A) HR >100 bpm (1.5 points); B) Signs of DVT (3 points); C) Active malignancy (1 point); D) Previous PE/DVT (1.5 points); E) PE most likely diagnosis (3 points)	USMLE Step 2 CK	B, E (both 3 points)
2	Diagnosis	A 38-year-old man after a long flight has Wells score 1.5, D-dimer 450 ng/mL. What is the next step?	A) CT angiography; B) Discharge; C) Apply age-adjusted D-dimer; D) Empirical anticoagulation; E) Venous duplex ultrasound	USMLE Step 2 CK	C
3	Diagnosis	67-year-old female with COPD, Geneva 6 (intermediate), D-dimer 1200, CT indeterminate. Next diagnostic step?	A) Repeat CT; B) V/Q scan; C) Venous duplex; D) Empirical anticoagulation; E) Pulmonary angiography	ABIM Board	C
4	Diagnosis	72-year-old male, PESI variables yield total 172 points. What is his risk class?	A) 172 – Class V; B) 152 – Class IV; C) 132 – Class III; D) 112 – Class III; E) 92 – Class II	USMLE Step 2 CK	A
5	Diagnosis	45-year-old female with antiphospholipid syndrome, persistent CT filling defect after 6 months. Most likely cause?	A) Recurrent PE; B) Chronic organized thrombus; C) Pulmonary artery sarcoma; D) Artifact; E) AV malformation	ABIM Board	B
6	Management	28-year-old female with stable PE, Hestia criteria for outpatient care. Which is a contraindication?	A) Lobar PE; B) HR 90–100; C) Mild troponin elevation; D) Patient preference; E) Weight >100 kg	ACCP Board	C
7	Diagnosis	58-year-old male, post-surgery, prior DVT, HR 88. What is modified Geneva score?	A) 3; B) 5; C) 7; D) 2; E) 9	USMLE Step 2 CK	B
8	Diagnosis	62-year-old male, Wells 7 (high), D-dimer 380 (negative). Next step?	A) Discharge; B) CT angiography; C) Repeat D-dimer; D) V/Q scan; E) Start anticoagulation	USMLE Step 2 CK	B
9	Diagnosis	55-year-old female, CKD stage 4, contrast allergy. Best diagnostic strategy?	A) Non-iodinated CT; B) V/Q scan; C) Venous duplex; D) MR angiography; E) Empirical anticoagulation	ABIM Board	B
10	Diagnosis	Which statement on D-dimer is most accurate?	A) High sensitivity and specificity; B) Age-adjusted cutoff improves specificity; C) Elevated confirms PE; D) Useful in high probability; E) Negative rules out PE in all cases	USMLE Step 2 CK	B
11	Treatment	48-year-old female, post-hip replacement PE. Recommended minimum duration of therapy?	A) 6 weeks; B) 3 months; C) 6 months; D) 12 months; E) Indefinite	ACCP Board	B
12	Treatment	60-year-old male with new PE, CrCl 78. Which initial regimen is evidence-based?	A) Warfarin only; B) Rivaroxaban; C) Apixaban; D) LMWH bridge; E) B, C, D	ABIM Board	E
13	Treatment	54-year-old female with metastatic pancreatic cancer, PE. Best regimen for cancer-associated VTE?	A) Warfarin; B) LMWH; C) Rivaroxaban; D) Apixaban; E) All equal	ACCP Board	B
14	Treatment	52-year-old male on rivaroxaban 6 weeks for PE. Which monitoring advice?	A) INR monthly; B) Anti-Xa monthly; C) No routine coagulation; check renal and CBC; D) Weekly PT/aPTT; E) None at all	ABIM Board	C
15	Treatment	65-year-old female on UFH day 5, platelets drop 245→98k. HIT suspected. What next?	A) Continue UFH; B) Switch to LMWH; C) Stop heparin, start argatroban/bivalirudin; D) Stop heparin, start warfarin; E) Stop all anticoagulation	USMLE Step 2 CK	C
16	Treatment	42-year-old male with submassive PE with RV dysfunction. Thrombolysis guideline?	A) All submassive; B) Case-by-case; C) Prefer catheter-directed always; D) Contraindicated; E) Prophylactic use	ACCP Board	B
17	Treatment	58-year-old male with PE and atrial fibrillation. How to anticoagulate?	A) Warfarin + apixaban; B) Single regimen covers both; C) ASA + DOAC; D) Warfarin + ASA; E) Two regimens separately	ABIM Board	B
18	Treatment	35-year-old female, 28 weeks pregnant, PE. Best regimen?	A) Warfarin; B) LMWH during pregnancy; switch postpartum; C) Apixaban; D) Rivaroxaban; E) UFH entire pregnancy	USMLE Step 2 CK	B
19	Treatment	70-year-old male with PE, CrCl 25. Which statement true?	A) Warfarin safe; B) Rivaroxaban contraindicated; C) Apixaban dose reduce; D) Enoxaparin adjust; E) All correct	ACCP Board	E
20	Complications	Which findings indicate increased PE mortality risk?	A) Elevated troponin; B) Elevated BNP; C) RV dysfunction; D) Hypotension/shock; E) All above	USMLE Step 2 CK	E
21	Complications	Which factors predict highest recurrence after stopping anticoagulation?	A) Male; B) Unprovoked; C) High D-dimer post-stop; D) Residual thrombosis; E) All, especially A+B	ABIM Board	E
22	Management	45-year-old female with massive PE, hypotensive. Initial management?	A) Systemic thrombolysis; B) Anticoagulate, reassess; C) Surgical embolectomy; D) Catheter-directed thrombolysis; E) Supportive care only	ACCP Board	A
23	Management	58-year-old male with PE and DVT on anticoagulation. Need IVC filter?	A) Always; B) Only if contraindicated/failure; C) Routine retrievable; D) Improves survival; E) Permanent preferred	ABIM Board	B
24	Complications	52-year-old male with submassive PE. Absolute contraindication to thrombolysis?	A) Age >75; B) Stroke 6 months ago; C) Major surgery 2 weeks; D) BP 185/105; E) Already anticoagulated	USMLE Step 2 CK	B
25	Management	67-year-old female with massive PE and RV failure, hypotensive despite fluids. Best hemodynamic management?	A) Aggressive fluids; B) Cautious fluids; C) Immediate high-dose inotropes; D) Paracentesis; E) Intubation with PEEP	ACCP Board	B
